# First case report of pulmonary and cutaneous nocardiosis caused by *Nocardia mexicana* in Iran

**DOI:** 10.1099/acmi.0.000016

**Published:** 2019-04-23

**Authors:** Darioush Shokri, Tahereh Motalebirad, Morteza Jafarinia, Davood Azadi, Kazem Ghaffari

**Affiliations:** 1 Infectious Diseases and Tropical Medicine Research Center, Isfahan University of Medical Sciences, Isfahan, Iran; 2 Department of Microbiology, School of Medicine, Isfahan University of Medical Sciences, Isfahan, Iran; 3 Department of Immunology, School of Medicine, Isfahan University of Medical Sciences, Isfahan, Iran; 4 Molecular and Medicine Research Center, Arak University of Medical Sciences, Arak, Iran; 5 Department of Laboratory Sciences, Khomein University of Medical Sciences, Khomein, Iran

**Keywords:** *Nocardia mexicana*, Pulmonary infection, cutaneous wound, Linezolid

## Abstract

**Background:**

*
Nocardia* are aerobic partially acid-fast bacteria that are environmentally ubiquitous. This group of bacteria causes a rare bacterial infection of either the lungs (pulmonary) or body (systemic) that usually affects immunocompromised individuals. *Nocardia mexicana* was first isolated in 2004 from a patient with chronic bronchitis. However, there have been few reports on the clinical significance of this organism up to now. We herein report the first cases of *N. mexicana* in patients with pulmonary and cutaneous infection from Iran.

**Case presentation:**

A 57-year-old man was admitted to hospital due to a cutaneous wound on his left foot, fever, weakness, persistent cough and chest pain. At first, due to clinical examination and laboratory test, the patient was diagnosed as having tuberculosis. However, PCR of *Mycobacterium tuberculosis* was negative from broncho-alveolar lavage (BAL) samples. Direct PCR of BAL was performed for this patient and according to the clinical examinations and microbiological evaluations; the micro-organism was identified as *N. mexicana* and was isolated from both BAL and the wound. Finally, the patient was treated with linezolid and amikacin.

**Conclusion:**

The infections, with actinomycetes such as *Nocardia *, are easily neglected or misdiagnosed due to the fastidious nature of the organism and the inadequate microbiological experience of laboratories in the hospitals of developing countries. This case shows that hospitals should consider a better laboratory protocol to deal with the clinical cases in which fastidious organisms, and in particular *Nocardia *, are involved.

## Introduction

Nocardiosis is a rare, opportunistic bacterial infection caused by *Nocardia* species that predominantly affects the respiratory tract of patients [1]. *Nocardia* was first described by [2] and classified as an aerobic Gram-positive, slow-growing, filamentous hypha-like bacterium and member of the genus *Nocardia *, order Actinomycetales [3]. The genus *Nocardia* includes >80 species, of which >30 have been shown to cause disease in humans. *Nocardia* species are found free-living (normal flora) in environmental resources such as water, soil, dust, plants and animals [1, 3]. Moreover, this bacterium can also live in soil and still water and enter the human body through dust respiration and traumatic inoculation and give rise to infections such as lung infection, septicemia, chronic bronchitis and brain abscess [4, 5]. Immunocompromised patients, such as those with cancer and diabetes, AIDS, connective tissue disorders, and those who receive immunosuppressive drugs or transplants are among the main hosts of *Nocardia* infections [6]. However, isolated cases have been reported in immunocompetent hosts [5, 7].

*N* *. mexicana* is Gram-positive, slow-growing and partially acid-fast bacilli, it was first isolated by Rodríguez-Nava *et al*. from human mycetomas [8]. To date, this micro-organism has been reported from pulmonary infections, mycetoma and arthritis [9–11]. Amongst the *Nocardia* species, *N. mexicana* is not frequently isolated, and infections caused by this species are rarely reported. In the present study, we report the first case of pulmonary and cutaneous wound infection in a patient from Isfahan, Iran.

### Patient introduction

**Fig. 1.**
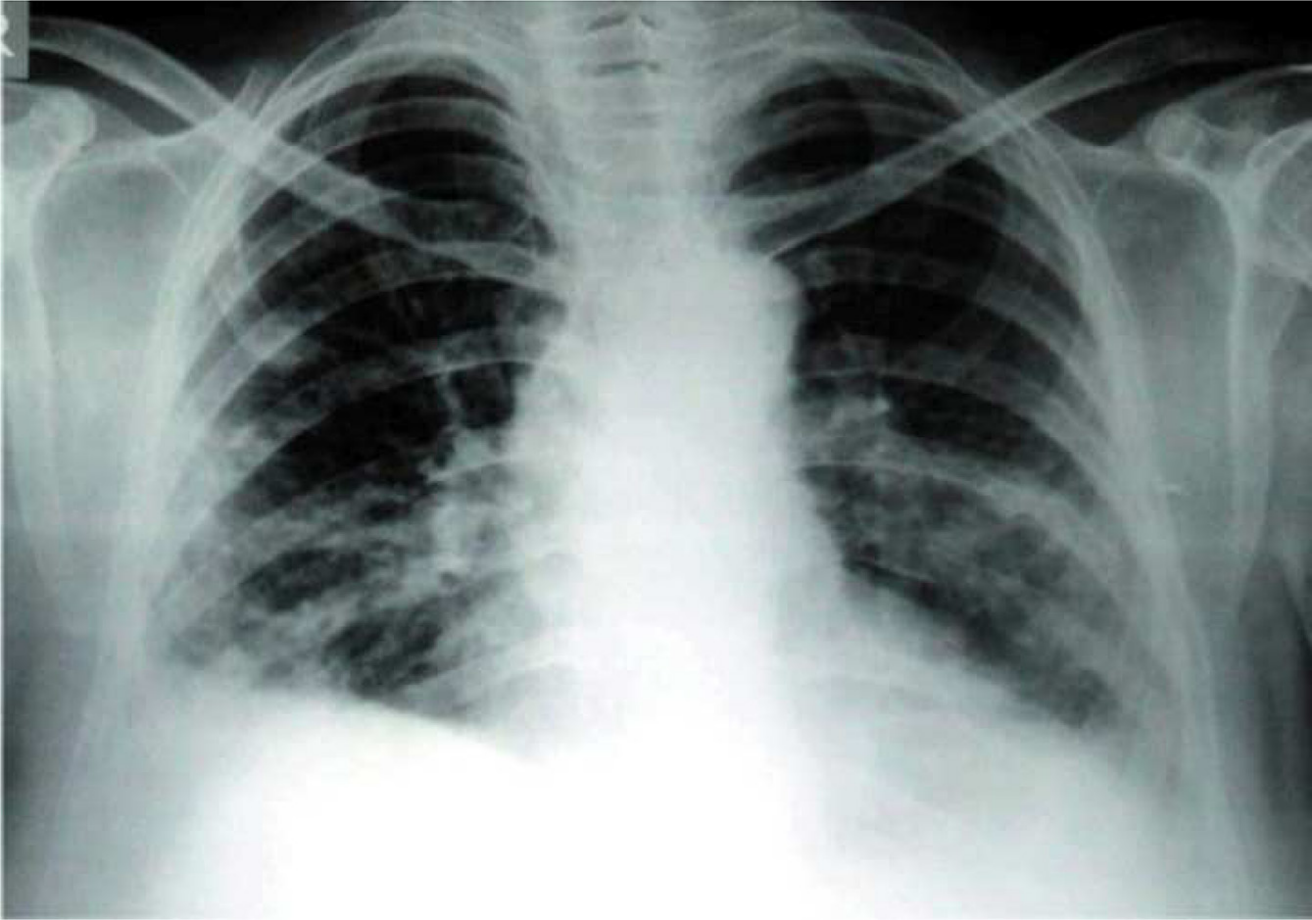
Chest x-ray from the patient, showing nodular lesions across both lungs.

**Fig. 2.**
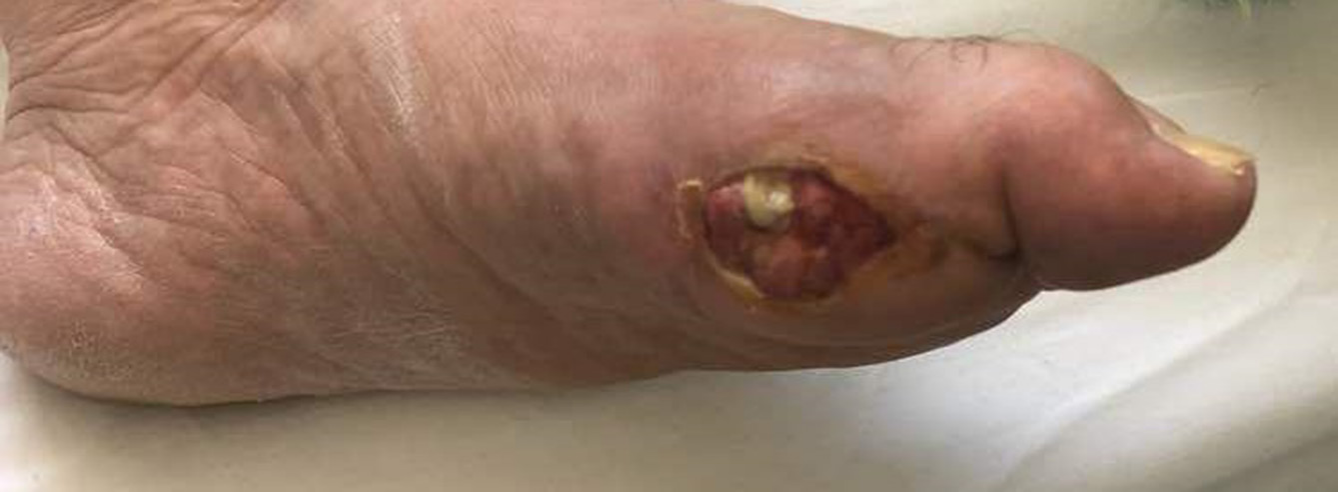
Cutaneous wound on the left foot of the patient.

A 57-year-old man with no history of human immunodeficiency virus (HIV) infection, immunodeficiency or consumption of immunosuppressive drugs was admitted to hospital in Isfahan, with symptoms including a 4-month history of a cutaneous wound on his left foot acquired during agriculture, persistent fever (39 °C), weakness, persistent non-productive cough and chest pain; a chest x-ray was taken and evaluation of radiological pictures showed nodular lesions across both lungs (Fig. 1); Purified protein derivative (PPD) test was negative and a erythrocyte sedimentation rate (ESR) of 57 mm h^−1 ^was present. The patient was suspected of having tuberculosis, *Staphylococcus aureus* was isolated from his foot wound and anti-tuberculosis drugs were initiated. However, the patient was not treated after 2 weeks and his cutaneous wound had not improved and developed into a productive large lesion, expelling yellow-wan pus similar to mycetoma (Fig. 2).

## Methods

In our laboratory, a biopsy of the cutaneous wound and a broncho-alveolar lavage (BAL) sample was obtained from the patient, cultured on blood agar and chocolate agar, Löwenstein–Jensen, Sauton's agar (a litre contains 100 g of glucose, 15 g agar and 20 ml of 50% yeast extract), Sabouraud Dextrose Agar (SDA) and anaerobic culture media and were incubated at a temperature of 35±2 °C for 3–4 weeks.

The isolates were identified by culture and phenotypic features included direct smear analysis by Gram and partial acid-fast staining, culture on blood agar and Sauton’s agar and incubation at 37 and 45 °C. Biochemical tests such as casein, tyrosine, xanthine, gelatin, aesculin and adenine degradations, urease and citrate production were used to identify the isolate. The drug sensitivity of the isolate was evaluated by the broth microdilution method, based on criteria indicated by the Clinical and Laboratory Standards Institute (CLSI) M24-A2.

### Molecular identification

Chromosomal DNA from identified isolates and direct (BAL) sample was extracted using a simple boiling method. In brief, several colonies of bacteria were added to 200 ml Tris EDTA buffer, boiled for 15 min and centrifuged at 11 180 ***g*** for 10 min. The supernatant was transferred to another sterile microtube and centrifuged at 20 000 ***g*** for 15 min. Precipitated DNA was resuspended in 50 µl Milli-Q water and stored at 20 °C.

**Fig. 3.**
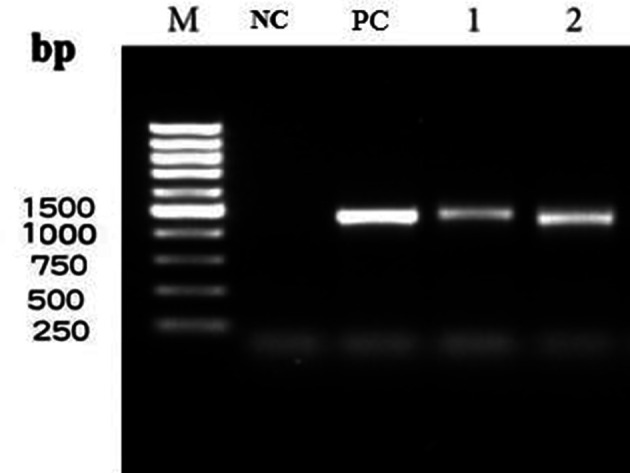
Agarose gel electrophoresis of 16SrRNA PCR amplification of the Iranian isolates; NC: negative control, PC: positive control (* N. asteroides *), 1: DSH41 isolate from culture, 2: direct BAL samples.

The isolates identified phenotypically as *Nocardia* were further analysed to the genus and species levels using a panel of molecular tests that included a genus-specific PCR based on a 596 bp region of the 16S rRNA, as recommended by Laurent [12], followed by the amplification and direct analysis of almost complete 16S rRNA sequencing for species identification, as described by Roth [13] (Fig. 3). Sequencing was performed by Bioneer (South Korea), and the sequence data received were aligned manually with existing sequences of *Nocardia* retrieved from the GenBank database and analysed using the Blast program in GenBank and the jPhydit program [14].

## Result

**Fig. 4.**
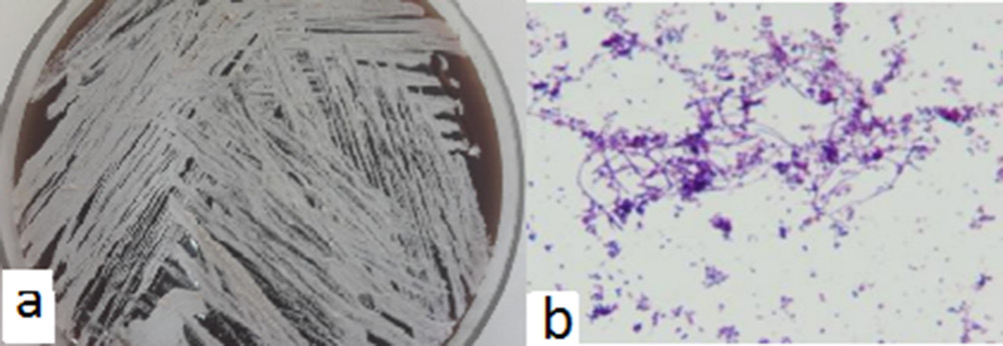
(a) Small chalky white non-haemolytic colonies of the isolate on blood agar medium. (b) Microscopic morphology of the isolate; filamentous branching Gram-positive rods (×1000).

The partial acid-fast and Gram staining of the colonies indicated Gram-positive rods with branched filaments and colony morphology, pigment production, growth rate (<7 days) and resistance to lysozyme, resembling species of the genus *Nocardia* (Fig. 4). After 48 h of incubation at 37 °C in an aerobic atmosphere, small, rough, dry and non-haemolytic chalky white colonies emitting an earthy odour were observed on blood agar. The isolates were positive for catalase production, aesculin hydrolysis, and decomposition of adenine, hypo-xanthine and urease and was negative for casein, tyrosine, citrate and xanthine. The antibiotic sensitivity test showed that the isolate was susceptible to ciprofloxacin, clarithromycin, ceftriaxone and linezolid and resistant to TMP/SMX and minocycline.

The PCR amplification of a genetic marker based on a 596 bp region of the 16S rRNA confirmed the identity of the isolate as a member of the genus *Nocardia *. The 16S rRNA gene sequence of the isolate DSH41 had nucleotide signatures of *Nocardia* genus [at positions including 70–98 (U-A), 139–224 (G-C), 843 (C), 1008–1021 (C-G), 1189 (C), 1244–129 (C-G) and 1308–1329 (C-G)], which showed 100% similarities with the corresponding sequence of *N. mexicana* strain DSM 44952. The GenBank accession number of the 16S rRNA sequence of the Iranian isolate is LC152749.

**Fig. 5.**
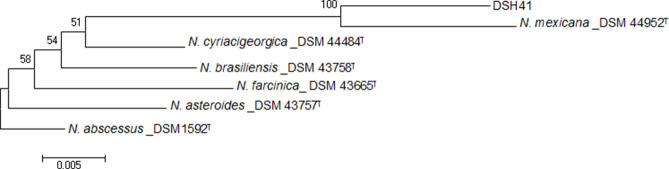
The 16S rRNA sequence based phylogenetic tree for the Iranian Nocardia isolate and nearest validated species of *Nocardia* using the neighbour-joining method.

The relationship between our isolates and the established standard species of *Nocardia* was supported by a high bootstrap value in the phylogenetic tree based on the 16S rRNA gene depicted by mega 7 software (Fig. 5).

Furthermore, results of direct PCR of BAL sample and sequence analysis of the bacterial isolate from foot wound that belonged to *N.mexicana,* which is show that the infection could be disseminated. After clinical and laboratory diagnosis of patients, the physicians was supposed that disseminated infection of *N. mexicana* for the patients; subsequently, Blood sample and brain computed tomography (CT) were obtained from him; but brain CT scan was normal and blood culture was negative.

The patient was initiated with linezolid and amikacin for 6 months; his respiratory symptoms recovered after 3 weeks and the foot wound gradually improved after a few weeks.

## Discussion

In the current study, we report a novel contemporary case report of both pulmonary and wound infection caused by *N. mexicana* from a farmer patient in Isfahan, Iran. To our knowledge, this report is the first of a *N. mexicana* infection in Iran and also in the primary literature of co-infection (pulmonary and wound) with *N. mexicana* in the world. This report showed evidence of the epidemiological distribution of *N. mexicana* in Iran; to date, this bacterium has only been reported from Mexico, the USA, Japan, Caucasus, Australia and Iran [9–11].

*N. mexicana* was isolated from mycetoma, cutaneous botryomycosis, pulmonary and cerebral abscess in humans and tenosynovitis and arthritis in animals [8]. *N. mexicana* is a closely related species to *N. terpenica ,* which was isolated from Japan [8].

*Nocardia* spp. are the cause of various infections, including pulmonary (as the most common), central nervous system (CNS), cutaneous and disseminated infection termed as nocardiosis; in Iran, according to the literature, the nocardiosis incidence rate was estimated at 1.88%, which is misdiagnosed with pulmonary tuberculosis, malignant tumours and fungal infection due to complexities in isolation technique, identification and similarities of symptoms with other diseases such as pulmonary tuberculosis [15]. Since clinical and radiological findings are not specific for nocardial infections, accurate diagnosis based on clinical manifestations and radiological images is impossible. Tuberculosis is endemic in Iran; therefore, pulmonary nocardiosis is often misdiagnosed as pulmonary tuberculosis. Moreover, *Nocardia* spp. are relatively slow-growing bacteria, which may be ignored due to its slow growth. Therefore, partial acid-fast staining, paraffin-baiting method and resistance to lysozyme are the most important phenotypic method for identifying Nocardia infections [16]. Nowadays, molecular techniques have improved diagnosis of nocardiosis and revolted classification of *Nocardia* species [16].

*Nocardia* spp. are partially acid-fast bacteria, which are saprophytes as normal-flora in soil and can invade the body caused by cutaneous inclusion such as the present case; nocardiosis infection usually occurred in immune-deficient patients but there are unusual clinical reports of nocardial infection in immune-competent people such as the present case involving a farmer with pulmonary and wound nocardiosis caused by uncommon *N. mexicana *.

Review of the literature showed that Kuchibiro *et al*. reported the first pulmonary nocardiosis caused by *N. mexicana* from bronchial lavage fluid using Kinyon staining and 16S rRNA, *gyrB*, *rpoB*, *secA1* and *hsp65* direct sequencing method from the patient with a previous history of bronchiectasis. Finally, the patient was cured by amikacin and linezolid [8]. DeWitt and his colleagues also published the first report of botryomycosis in a Spanish patient with a sculp cutaneous wound, which was treated followed by linezolid and amoxicillin-clavulanic acid [17]. The first case of *N. mexicana* infection in animals was reported by Owen *et al*. from Australia. The isolate was resistant to trimethoprim-sulphamethoxazole (TMP/SMX) and the animal was treated by recommendation of co-trimoxazole and ceftiofur [11].

All of the reports suggested that *N. mexicana* was resistant to TMP/SMX; although TMP-SXT is the first choice of treatment of nocardiosis; this problem was confirmed by American Thoracic Society (ATS) guidelines and Wallace studies where species identification is necessary to guide appropriate antibiotic therapy. Furthermore, Moylett *et al*. declared that all clinically strains of Nocardia were susceptible to linezolid; therefore, it seems that linezolid is the best alternative treatment of nocardiosis, particularly *N. mexicana* infection.

### Conclusion

We reported the first case of pulmonary and cutaneous wound nocardiosis caused by *N. mexicana* from Isfahan, Iran. Given that the complexities in diagnosis of nocardial infection, molecular methods such as 16S rRNA direct sequencing is an appropriate method for identification of nocardiosis. Moreover, *Nocardia* species should be identified to the species level for final diagnosis and suitable treatment and linezolid is the best choice of treatment of nocardiosis such as *N. mexicana* infections.

## References

[1] Minero MV, Marín M, Cercenado E, Rabadán PM, Bouza E (2009). Nocardiosis at the turn of the century. Medicine.

[2] Nocard E (1888). Note sur la Maladie des boeufs de la Guadeloupe connuesous Le nom de farcin. Ann Inst Pasteur.

[3] Saubolle MA, Sussland D (2003). Nocardiosis: review of clinical and laboratory experience. J Clin Microbiol.

[4] Kontoyiannis DP, Ruoff K, Hooper DC (1998). *Nocardia bacteremia*: report of 4 cases and review of the literature. Medicine.

[5] Budzik JM, Hosseini M, Mackinnon Jr AC, Taxy JB (2012). Disseminated *Nocardia farcinica*: literature review and fatal outcome in an immunocompetent patient. Surg Infect.

[6] Ambrosioni J, Lew D, Garbino J (2010). Nocardiosis: updated clinical review and experience at a tertiary center. Infection.

[7] Kim S, Lee KL, Lee DM, Jeong JH, Moon SM (2014). *Nocardia* brain abscess in an immunocompetent patient. Infect Chemother.

[8] Rodriguez-Nava V, Couble A, Molinard C, Sandoval H, Boiron P (2004). *Nocardia mexicana* sp. nov., a new pathogen isolated from human mycetomas. J Clin Microbiol.

[9] Raby E, Hiew V, Arthur I (2016). A case of *Nocardia mexicana* cerebral abscess highlights deficiencies in susceptibility testing and the utility of direct molecular identification. Pathology.

[10] Ikeda T, Nakanishi H, Morishita Y, Houdai K, Ito J (2016). First case report of pulmonary nocardiosis caused by *Nocardia mexicana*. JMM case reports.

[11] Owen H, Buckle K, Olm J, Leitner M, Pandey S (2015). Isolation of *Nocardia mexicana* from focal proliferative tenosynovitis and arthritis in a steer. Aust Vet J.

[12] Laurent FJ, Provost F, Boiron P (2000). Rapid identification of clinically relevant *Nocardia* species to genus level by 16S rRNA gene PCR. J Clin Microbiol.

[13] Roth A, Andrees S, Kroppenstedt RM, Harmsen D, Mauch H (2003). Phylogeny of the genus *Nocardia* based on reassessed 16S rRNA gene sequences reveals underspeciation and division of strains classified as *Nocardia asteroides* into three established species and two unnamed taxons. J Clin Microbiol.

[14] Jeon YS, Chung H, Park S, Hur I, Lee JH (2005). jPHYDIT: a JAVA-based integrated environment for molecular phylogeny of ribosomal RNA sequences. Bioinformatics.

[15] Hashemi-Shahraki A, Heidarieh P, Bostanabad SZ, Hashemzadeh M, Feizabadi MM (2016). Genetic diversity and antimicrobial susceptibility of *Nocardia* species among patients with nocardiosis. Sci Rep.

[16] Brown-Elliott BA, Brown JM, Conville PS, Wallace RJ (2006). Clinical and laboratory features of the *Nocardia* spp. based on current molecular taxonomy. Clinl microbiol rev.

[17] DeWitt JP, Stetson CL, Thomas KL, Carroll BJ (2018). Extensive cutaneous botryomycosis with subsequent development of *Nocardia*-positive wound cultures. J Cutan Med Surg.

